# Immune-related adverse events and the balancing act of immunotherapy

**DOI:** 10.1038/s41467-022-27960-2

**Published:** 2022-01-19

**Authors:** Michael Conroy, Jarushka Naidoo

**Affiliations:** 1 Beaumont RCSI Cancer Centre, Dublin, Ireland; 2 Beaumont Hospital, Dublin, Ireland; 3 RCSI University of Health Sciences, Dublin, Ireland; 4 Sidney Kimmel Comprehensive Cancer Centre at Johns Hopkins University, Baltimore, MD USA

**Keywords:** Cancer immunotherapy, Tumour immunology, Cancer immunotherapy, Prognostic markers, Adverse effects

## Abstract

The benefit from immune checkpoint inhibitors is tempered by immunologic toxicities, which involve diverse organs, have varying biology, onset time, and severity. Herein, we identify important areas of controversy and open research questions in the field of immune-related toxicity.

## Immune checkpoint inhibitors and immune-related adverse events in cancer therapy

The oncology field has been historically dominated by cytotoxic chemotherapy. Immune checkpoint inhibitors (ICI) represent a significant step forward in the clinical practice of medical oncologists. With responses across several tumour types, immunotherapy has resulted in durable responses for selected patients and even a tentative re-introduction of the word ‘cure’^1^.

However, it is not only the efficacy of ICIs that distinguishes them from chemotherapy. These treatments are also linked to a new cadre of side effects, termed immune-related adverse events (irAEs). Immune-related toxicities are autoimmune conditions that can affect any organ in the body after ICI administration, with natural histories that are distinct from their de novo autoimmune disease counterparts. Thus, these toxicities represent a varied challenge in clinical practice and a steep learning curve to diagnose and manage. Rather than managing the familiar nausea, immunosuppression and anaemia, we now face underactive pituitary glands, inflamed bowel segments and hepatitis (Table 1). A decade on from the first immunotherapy approval, how much do we know about these cryptic harms and how do we balance their risk with the undeniable benefits of immunotherapy?

**Table 1:** Prevalence of irAEs and high-grade irAEs in major immunotherapy clinical trials.

Selected phase II and III trials	Immune-related adverse events		
	All %	High grade %	Top three most frequent (% of patients)
PEMBROLIZUMAB			
NSCLC			
NCT02142738—Keynote 024	29	10	Hypothyroidism (9), Hyperthyroidism (8), Pneumonitis (6)
NCT01905657—Keynote 010	20	5	Hypothyroidism (8), Hyperthyroidism (5), Pneumonitis (5)
Melanoma			
NCT03142334—Keynote 564	35	9	Hypothyroidism (21), Hyperthyroidism (12), Pneumonitis (2)
NCT01866319—Keynote 006	27	10	Hypothyroidism (11), Hyperthyroidism (5), Colitis (3)
NCT02362594—Keynote 054	37	7	Hypothyroidism (14), Hyperthyroidism (10), Vitiligo (5)
Urothelial			
NCT02256436—Keynote 045	17	5	Hypothyroidism (6), Hyperthyroidism (4), Pneumonitis (4)
NCT02335424—Keynote 052	26	10	Hypothyroidism (11), Pneumonitis (5), Hyperthyroidism (3)
NIVOLUMAB			
Melanoma			
NCT01721746—Checkmate 037	NR	NR	Pruritus (22), Diarrhoea (18), Rash (13)
NCT02388906—Checkmate 238	NR	NR	Diarrhoea (24), Pruritus (23), Rash (20)
NCT01721772—Checkmate 066	60	7	Pruritus (17), Diarrhoea (16), Rash (15)
Urothelial			
NCT02387996—Checkmate 275	NR	NR	Dermatologic (24), Gastrointestinal (13), Hepatic (6)
ATEZOLIZUMAB			
NSCLC			
NCT02409342—Impower 110	40	7	Hepatitis (16), Rash (15), Hypothyroidism (9)
NCT02486718—Impower 010	52	8	Rash (18), Hepatitis (17), Hypothyroidism (17)
Urothelial			
NCT02108652—IMvigor210	12	7	Rash (3), ALT increase (2), Rhabdomyolysis (2)
DURVALUMAB			
NSCLC			
NCT02125461—PACIFIC	24	3	Pneumonitis (11), Hypothyroidism (9), Hyperthyroidism (3)
Urothelial			
NCT02516241—DANUBE	18	6	Diarrhoea (7), Rash (7), Hypothyroidism (6)
IPILIMUMAB			
Melanoma			
NCT00094653—Hodi et al. 2010.	61	15	Diarrhoea (32), Pruritus (24), Rash (20)
NCT01866319—Keynote 006	19	12	Colitis (7), Hyperthyroidism (2), Hypothyroidism (2)
NCT01844505—Checkmate 067	NR	NR	Pruritus (36), Diarrhoea (34), Rash (22)
NIVOLUMAB/IPILIMUMAB			
NSCLC			
NCT02477826—CheckMate 227	NR	NR	Skin (34), Endocrine (24), gastrointestinal (18)
Melanoma			
NCT01844505—CheckMate 067	NR	NR	Diarrhoea (45), Pruritus (36), Rash (30)
Renal cell carcinoma			
NCT02231749—Checkmate 214	79	48	Pruritus (31), Diarrhoea (28), Rash (23)
DURVALUMAB/TREMELIMUMAB			
NSCLC			
NCT02453282—MYSTIC	28	11	Hypothyroidism (8), Pneumonitis (7), Diarrhoea (5)
Urothelial			
NCT02516241—DANUBE	37	17	Diarrhoea (22), Rash (15), Hypothyroidism (7)

*NSCLC* non-small cell lung cancer, *NR* not reported, *ALT* alanine aminotransferase.

There are some fundamental principles we now understand about irAEs. Immune-related toxicities vary in terms of their time of onset, severity, and underlying biology. They affect a broad range of organs and thus require a tailored management approach. They can occur at any time during a patient’s treatment course, most commonly in the first 3 months of treatment, but sometimes long after ICI has been discontinued. Second, despite this heterogeneity of presentation, the management of irAEs is centred around treatment with glucocorticoids. Most symptomatic irAEs (except endocrinopathies) are managed with several weeks of glucocorticoid treatment, with good effect. Third, while most irAEs resolve, some become chronic and may require lifelong therapy such as hormonal supplementation or immunosuppression.

More importantly, there are several aspects of irAEs that we do not understand and which are the source of several controversies in the field.

Several high-impact publications have demonstrated a positive association between the development of irAEs and anti-tumour responses to ICIs, across tumour types^2,3^. In addition, we have seen incremental gains in survival outcomes as patients develop numerically more irAEs^4^. It is hypothesised that both the anti-tumour response and the development of irAEs are representative of a robust immune reaction, where self-reactive T cells infiltrate both tumours and the organs that develop irAEs. However, other research has suggested poorer outcomes among patients with early or specific irAEs^5^. The studies which identify these associations, while interesting and somewhat intuitive, have come under scrutiny due to challenges in adjudication and attribution of irAEs, and immortal-time bias^6^.

Other uncertainties relate to diagnostic challenges. For example, pneumonitis is frequently occult on chest radiography and may only be identified on CT^7^. Even with cross-sectional imaging, findings can be diverse and the diagnosis may elude specialists without experience in the area. Similarly, immune-mediated nephritis is often difficult to distinguish histologically from other causes of kidney injury, including renal toxicity from chemotherapy drugs. Thus, inconsistent criteria used for the diagnosis of certain irAEs have hampered progress regarding diagnostic certainty. This underscores a greater need for standardised multidisciplinary definitions, as evidenced by recent consensus papers on definitions, such as for neurological irAEs^8^.

Perhaps the greatest difficulties in the field of irAEs surround their management. Given that there may be similar immune mechanisms responsible that underpin both tumour control and irAEs, researchers were concerned initially that steroids or other immunosuppressive agents to control irAEs would also hinder tumour control. However, this does not appear to be the case in retrospective studies^9^. Conversely, patients who begin immunotherapy while receiving corticosteroids (for example, in the treatment of brain metastases) appear to have worse outcomes^10^. However, use of steroids in this setting may be a confounder that reflects underlying ill health rather than a risk factor itself for poorer response to immunotherapy. An additional concern when initiating immunotherapy is the approach to patients with known autoimmune disease, who may be at risk for a flare of their illness, and thus may not be offered ICIs. While data in this area is limited, it appears that patients with a history of autoimmune disease are no more likely than patients without such a history to experience severe irAE, and may have similar antitumor responses to ICIs^11^. However, they may be more likely to discontinue their ICI due to an irAE, or be at higher risk of an irAE specific to their condition (for example, colitis in a patient with a history of IBD). Even if they are not harmful, steroids are sometimes ineffective for irAEs. Steroid-refractory colitis, myocarditis^12^ and pneumonitis^13^ have been described, and are characterised by high mortality rates and significant uncertainty regarding optimal management strategies. Options available include intravenous immune globulin (IVIG) and Infliximab, a monoclonal antibody that targets the proinflammatory cytokine TNF-α. However, management decisions are often based on either expert opinion or retrospective data, rather than prospective trials.

## What does the future hold for patients on immunotherapy with respect to irAEs, and their treating teams?

From a preclinical perspective, attention is focused on the development and optimisation of preclinical models, to better elucidate irAE mechanisms that may tailor diagnosis and management. Murine models utilising therapeutics of non-mouse origin appear to be poorly representative of irAEs in humans. A recent example of promising research in this area is a genetic mouse model that closely recapitulates ICI myocarditis and can be used to investigate therapeutic interventions^14^. This model allowed investigators to explore the consequences of CTLA-4 and PD-1 loss, the interactions of these two genes, mechanisms of myocarditis, and the use of abatacept to mitigate the course of myocarditis.

Given the myriad of challenges in managing irAEs, the focus has shifted towards tools that prevent irAEs. An area of growing interest is the identification of biomarkers to predict irAEs. It is well established that the gut microbiome is altered in inflammatory bowel disease (IBD), but also that it can be associated with enhanced antitumour response to ICIs^15^. One hypothesis considers this phenomenon to be attributable to an abundance of anti-inflammatory species, such as *Faecalibacterium prausnitzii*, which promotes sequestration of regulatory T cells within the intestine. Regulatory T-cells express high levels of CTLA-4, and therefore may be inactivated by ipilimumab and allow effector T cell activation, causing both tumour response and colitis. More recently, evidence suggests a relationship between gut microbial composition and response to irAE treatments^16^. Based on this knowledge, faecal microbiota transplantation has been used successfully been used to treat ICI colitis in a case series^17^, and is currently being investigated in a phase I clinical trial (NCT04038619). Interest is now focused on whether modulation of the microbiome could be used to prevent irAEs. It has been demonstrated that in mice with CTLA-4-induced colitis, administration of *Bifidobacterium* resulted in significantly less weight loss without compromising the therapeutic efficacy of ICI^18^. This effect is thought to be attributable to modulation of the metabolic functions of regulatory T cells.

Other irAE research has focused on the role of human leucocyte antigen (HLA) genes, a complex of genes involved in regulating immune responses, that may be implicated in the development of particular autoimmune diseases, such as atopic dermatitis and IBD. Given the noted similarities between irAEs and autoimmune diseases, it is not surprising that specific HLA alleles have been associated with the development of irAEs, including colitis^19^. This suggests a role for HLAs in stratifying the risk of irAEs, identifying a group that requires more intensive monitoring. The above insights are being tested in a clinical trial (NCT04107311) that aims to prospectively investigate the role of the intestinal microbiome and autoimmune panels as predictors of ≥ Grade 2 irAEs requiring immunosuppression. In the future, large cohort studies investigating high-risk HLA alleles could also permit irAE risk profiling of patients in advance of ICI treatment, and allow physicians to adjust treatment plans, or intensity of surveillance, based on these risk models.

Regarding management, there are several clinical trials that aim to elucidate optimal treatment strategies for steroid-refractory irAEs. One example is a trial involving patients with steroid refractory pneumonitis who were randomly assigned to infliximab or IVIG alongside steroids (NCT04438382). Similarly, a phase I/II trial is underway that aims to compare the efficacy of infliximab vs. vedolizumab alongside corticosteroids for steroid-refractory colitis (NCT04407247).

In order to create an environment supportive of these innovations in irAE research and clinical care, several institutions have set up multidisciplinary immune-related toxicity teams^20^. These teams consist of a broad range of specialists representing irAE needs in high-volume institutions that provide a centralised irAE specialist service to ensure prompt recognition and treatment of these complications and a coordinated research enterprise.

These teams and their collective wisdom will ensure progress in irAE diagnosis and management, guided by insights from both clinical care and biospecimens obtained at key timepoints, such as baseline, time of irAE diagnosis and irAE resolution.

**Fig. 1:**
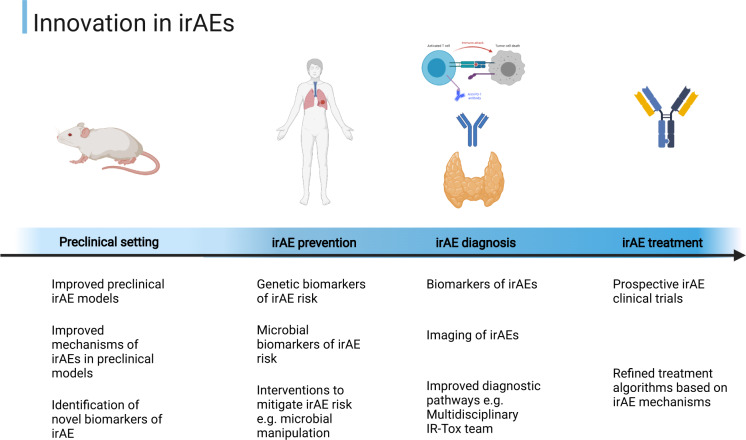
Innovation in immune-related adverse event research spans the preclinical setting (preclinical models exploring mechanisms and biomarkers), irAE prevention studies (based on genetic or microbial biomarkers and potential interventions), studies that focus on optimizing irAE diagnosis (based on biomarkers or imaging to improve diagnostic pathways), and lastly irAE treatment (prospective irAE trials to inform guidelines).

IrAEs present a unique challenge in modern oncology, and our understanding of them is evolving rapidly. There is growing consensus regarding their pathophysiology and management, but more work is needed to elucidate the relationships between irAEs and treatment outcomes, specific biomarkers that clinch an irAE diagnosis, the impact of tailored immunosuppression on ICI outcomes, and the management of steroid-refractory irAEs. In the near future, we are likely to see increasingly sophisticated preclinical models of irAEs, risk stratification with irAE biomarkers, and evidence based approaches for the management of irAE subsets such as steroid-refractory disease (Fig. 1). These advances will support optimal care for all patients receiving ICIs, guided by cross-pollination of the expertise and insights gained from multidisciplinary teams.
